# Bis[5-(anthracen-9-ylmeth­yl)-1,5,9-tri­aza­cyclododecan-1-ium] tetra­chlorido­zincate

**DOI:** 10.1107/S2414314625003566

**Published:** 2025-05-02

**Authors:** Yoshimi Ichimaru, Koichi Kato, Wanchun Jin, Masaaki Kurihara, Hiromasa Kurosaki

**Affiliations:** a Faculty of Pharmaceutical Sciences, Shonan University of Medical Sciences, 16-48, Kamishinano, Totsuka-ku, Yokohama, 244-0806, Japan; b College of Pharmacy Kinjo Gakuin University, 2-1723 Omori Moriyamaku Nagoya 463-8521 Japan; University of Antofagasta, Chile

**Keywords:** crystal structure, polyamine, [12]aneN_3_, anthracene

## Abstract

A crystalline salt comprising two monoprotonated polyamine ligands and one tetra­chloro­zincate(II) anion was prepared, (C_24_H_32_N_3_)_2_[ZnCl_4_], and its crystal structure was analyzed and compared with those of structurally related compounds bearing different macrocyclic frameworks and pendant arms. The protonated nitro­gen atoms engaged in intra­molecular hydrogen bonding with other nitro­gen atoms within the macrocyclic ring. In the crystal, the pendant anthracene groups participated in inter­molecular π–π and C—H⋯π inter­actions, contributing to crystal cohesion.

## Abstract 2

The structure of the title salt comprising two monoprotonated polyamine ligands and one tetra­chloro­zincate(II) anion was analyzed and compared with those of structurally related compounds bearing different macrocyclic frameworks and pendant arms. The protonated nitro­gen atoms engaged in intra­molecular hydrogen bonding with other nitro­gen atoms within the macrocyclic ring.

## Graphical abstract

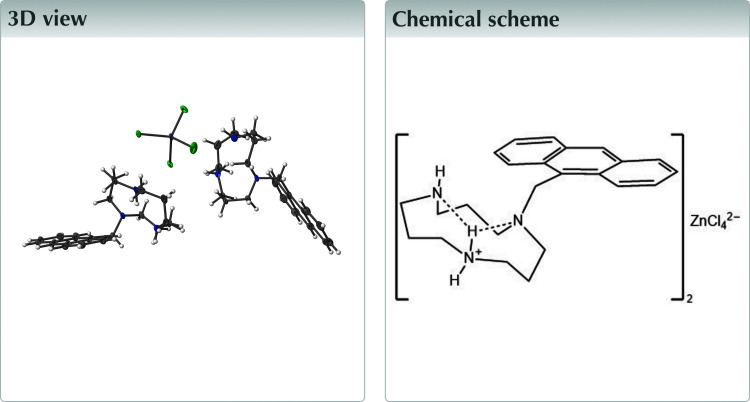

## Structure description

**Figure 1:**
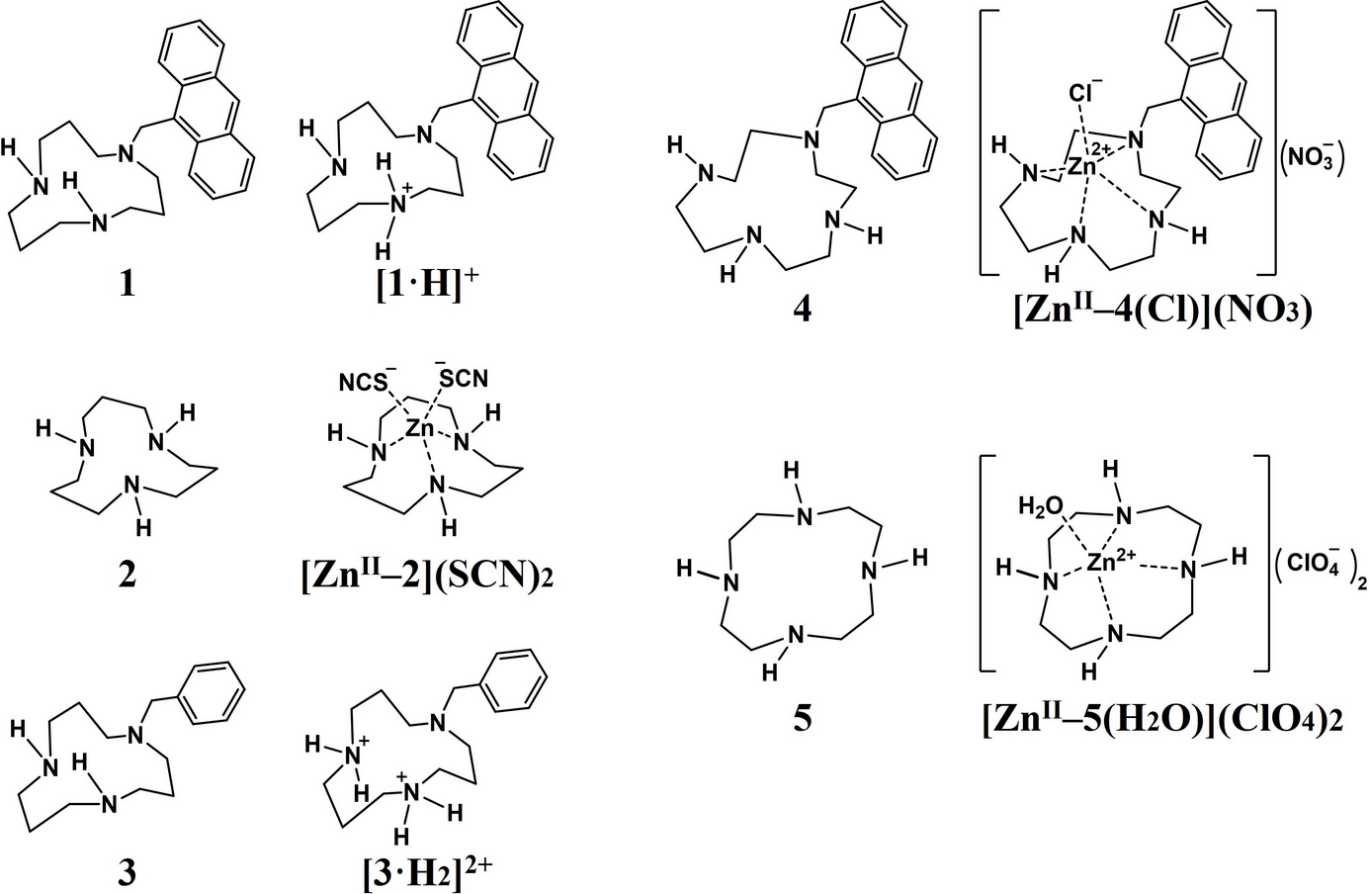
Chemical structures of the compounds referred to in the text.

The title complex is a salt comprising two monoprotonated cationic mol­ecules of 1-(anthracen-9-ylmeth­yl)-1,5,9-tri­aza­cyclo­dodecane (Ant-[12]aneN3, **1**) and a zinc tetra­chloride ion (see Fig. 1 ▸ for chemical structure). Ant-[12]aneN3 was designed as a ligand for DNA photocleavage by connecting the 1,5,9-tri­aza­cyclo­dodecane ([12]aneN3, **2**) moiety as the ligand for the zinc(II) ion with an anthracene mol­ecule as the photosensitizing species through a methyl­ene spacer (Ichimaru *et al.*, 2025 ▸). The zinc complex of [12]aneN3 is known as a model compound for the active center of the zinc enzyme carbonic anhydrase (Kimura *et al.*, 1990 ▸). In the crystal of [12]aneN3 with zinc thio­cyanate [Zn^II^–**2**](SCN)_2_, three nitro­gen atoms of the polyamine ring and two thio­cyanate ions are coordinated to the zinc(II) ion (Kimura *et al.*, 1992 ▸). In contrast with [Zn^II^–**2**](SCN)_2_, in which none of the nitro­gen atoms in the polyamine ring is protonated, one of the nitro­gen atoms of Ant-[12]aneN3 is monoprotonated ([**1**·H]^+^) in the title complex and the nitro­gen atoms of the polyamine ring are not chelated to the zinc(II) ion. Hubsch-Weber and Youinou synthesized 1-benzyl-1,5,9-tri­aza­cyclo­dodecane (**3**) and reported the crystal structure of the diprotonated cation ([**3**·H_2_]^2+^) formed *via* the reaction of **3** with Zn(ClO_4_)_2_·6H_2_O (Hubsch-Weber & Youinou, 1997 ▸). The crystal structures of ligands **1** and **3** bearing different pendant substituents exhibit inter­esting differences. The structural features of the title complex are described below in comparison with those of [**3**·H_2_]^2+^.

**Figure 2:**
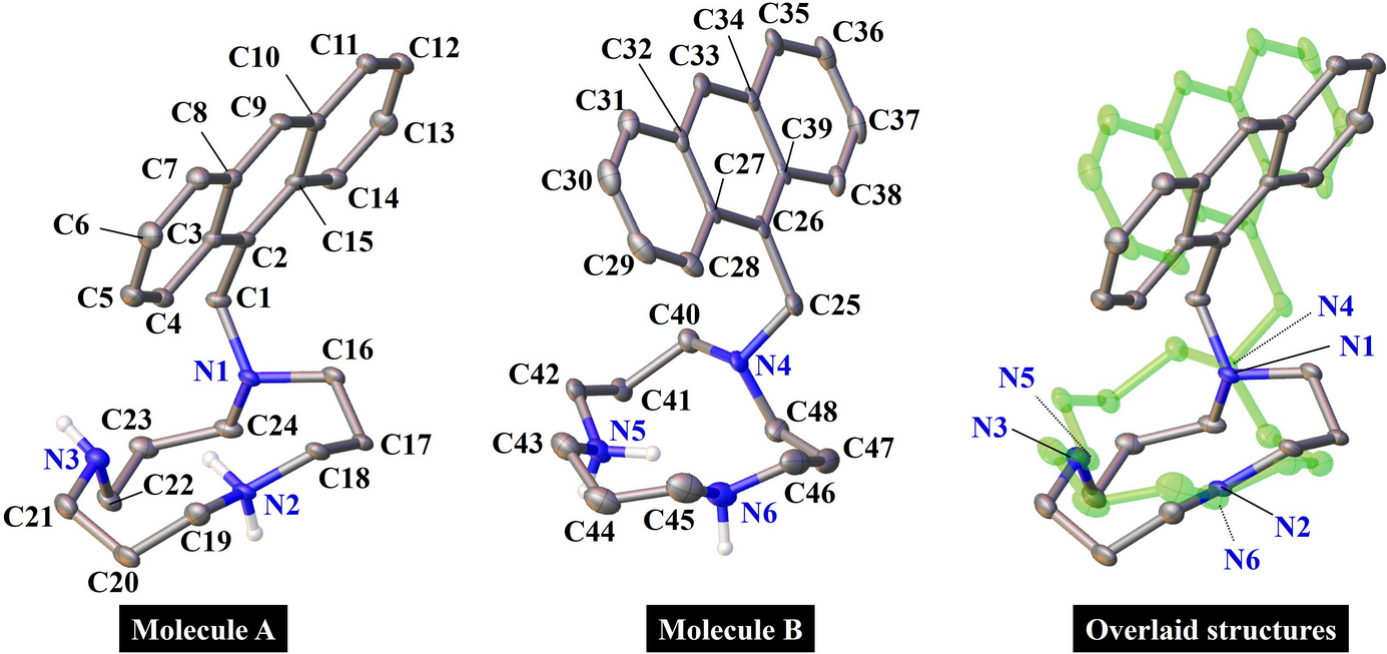
The cations (mol­ecules *A* and *B*) of the title compound, with displacement ellipsoids drawn at the 50% probability level, and their overlay diagram. Carbon-bound hydrogen atoms are omitted for clarity. In the overlay diagram, mol­ecule *B* is shown in green.

The asymmetric unit of the title complex contains two [**1**·H]^+^ mol­ecules (designated as mol­ecules *A* and *B*) and one tetra­chloro­zincate(II) (ZnCl_4_^2−^) anion, without solvent mol­ecules or additional counter-ions. The presence of ZnCl_4_^2−^, which is commonly formed in reactions involving zinc chloride (ZnCl_2_) (Al-Resayes *et al.*, 2017 ▸), confirms that both ligands are monoprotonated, consistent with the observed electron density (Fig. 2 ▸). Mol­ecules *A* and *B* are conformational isomers and contain three types of nitro­gen atoms: tertiary nitro­gen atoms (N1 and N4), protonated secondary nitro­gen atoms (N2 and N5), and nonprotonated secondary nitro­gen atoms (N3 and N6). The structural overlay diagram (Fig. 2 ▸) reveals that *A* and *B* are nonsuperimposable. Despite exhibiting opposite chiral conformations, *A* and *B* are not true enanti­omers owing to differences in the nitro­gen atom geometries and the centrosymmetric space group, *P*2_1_/*c*.

**Figure 3:**
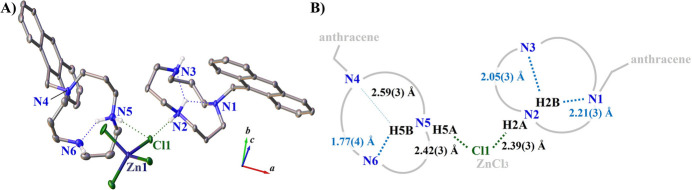
Inter­molecular and intra­molecular hydrogen-bond inter­actions of the title complex with displacement ellipsoids drawn at the 50% probability level. Carbon-bound hydrogen atoms are omitted for clarity. Hydrogen-bond inter­actions are shown as dotted lines. (*a*) *ORTEP*-style diagram showing the hydrogen bonds; (*b*) schematic showing the donor⋯acceptor distances of the hydrogen bonds.

A key factor influencing the nitro­gen atom geometry is the hydrogen-bonding network (Fig. 3 ▸). The H2*A* and H5*A* atoms bonded to protonated N2 and N5 form hydrogen bonds with Cl1. The distances between H2*A* and H5*A* and the acceptor, *i.e.*, N2—H2*A*⋯Cl1 and N5—H5*A*⋯Cl1, are nearly equal, *i.e.*, 2.39 (3) and 2.42 (3) Å, respectively (Table 1 ▸). The other H atoms hydrogen bonded to N2 and N5, respectively, are each oriented toward the inner pore of the macrocycle. The H2*B* and H5*B* atoms form hydrogen bonds with the nitro­gen atoms of the rings forming the pores. The hydrogen-bond geometries within the macrocycle are different for mol­ecules *A* and *B*. Specifically, the distances between these hydrogen atoms and the acceptor are 2.21 (3) Å (N2—H2*B*⋯N1) and 2.05 (3) Å (N2—H2*B*⋯N3) in mol­ecule *A* and 1.77 (4) Å (N5—H5*B*⋯N4) and 2.59 (3) Å (N5—H5*B*⋯N6) in mol­ecule *B*.

The nonprotonated secondary nitro­gen atom exhibits Lewis basicity, while the tertiary nitro­gen atom is slightly less basic owing to the presence of the pendant arm. Within the macrocycle of mol­ecules *A* and *B*, the nonprotonated secondary nitro­gen atom functions as a strong hydrogen-bonding acceptor group. In mol­ecule *B*, the tertiary nitro­gen atom does not function as a hydrogen-bonding acceptor. In **3**, there is no nonprotonated secondary nitro­gen atom. The basicity of the tertiary nitro­gen atom, which is substituted by a benzyl group as the pendant arm, is slightly impaired. Hence, none of the four hydrogen atoms bonded to the nitro­gen atoms are pointing outward in the macrocycle and hence do not form hydrogen bonds. Compared with those in mol­ecule *A* in the title compound, the distances between nitro­gen atoms in **3** are longer. Therefore, as mentioned above, nitro­gen protonation and hydrogen bond formation affect the geometry of nitro­gen atoms in the macrocycles.

**Figure 4:**
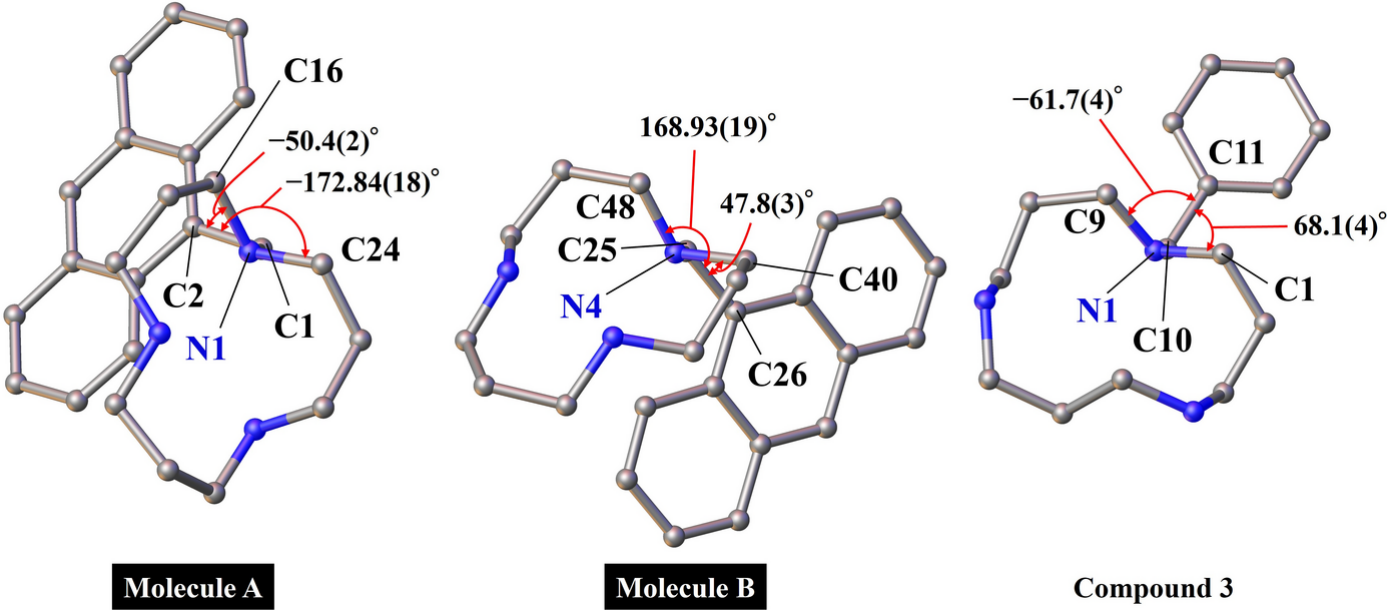
Dihedral angles of the macrocyclic structure and pendant arm i ball-and-stick models. H atoms and counter-anions are omitted for clarity.

The pendant anthracene moiety also affects the spatial arrangement of the macrocycle. As shown in Fig. 4 ▸, the benzyl group is directed away from the macrocyclic cavity in **3**. Meanwhile, in mol­ecules *A* and *B*, the anthracene moieties are positioned above the macrocyclic ring, partially overlapping the cavity. Although this orientation may be sterically disfavored in isolated mol­ecules, it facilitates inter­molecular π–π inter­actions in the crystal, contributing to structural cohesion.

**Figure 5:**
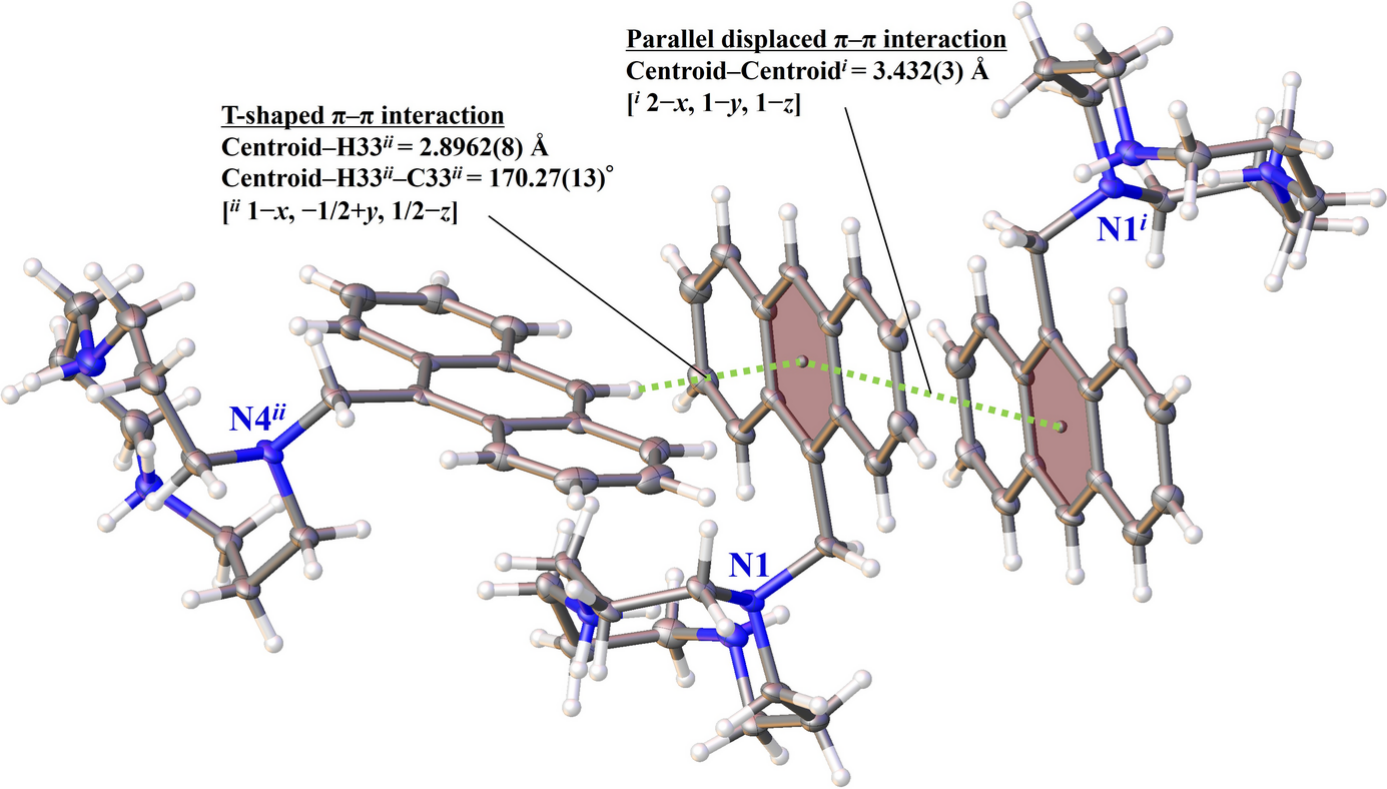
Schematic of the T-shaped and parallel displaced π–π inter­actions with displacement ellipsoids drawn at the 50% probability level. The tetra­chloro­zincate ions are omitted for clarity. The π–π inter­actions are shown as green dotted lines.

As shown in Fig. 5 ▸, two types of aromatic inter­actions are observed in the crystal: a parallel displaced π–π stacking inter­action and a T-shaped C—H⋯π inter­action. The face-to-face π–π stacking occurs between the anthracene rings, with a centroid-to-centroid distance of 3.432 (3) Å, which is typical for aromatic stacking inter­actions. Meanwhile, in the T-shaped inter­action, the C—H group from one anthracene unit (C33—H33) points toward the centroid of a neighboring aromatic ring, with a H⋯centroid distance of 2.90 Å and a C—H⋯centroid angle of 170°, respectively. These inter­actions further stabilize the packing structure by linking adjacent ligands *via* directional noncovalent forces.

Previously, we reported the crystal structure of the zinc(II) complex [Zn^II^–**4**(Cl)](NO_3_) containing 1-(anthracen-9-ylmeth­yl)-1,4,7,10-tetra­aza­cyclo­dodecane (Ant-[12]aneN4, **4**), which is an analog of Ant-[12]aneN3 (Ichimaru *et al.*, 2024 ▸). The polyamine ring of Ant-[12]aneN4 is 1,4,7,10-tetra­aza­cyclo­dodecane (cyclen, [12]aneN4, **5**), which chelates the zinc(II) ion with a counter-anion mol­ecule to form a five-coordinate structure (Ichimaru *et al.*, 2021 ▸), similar to that formed in the {[Zn^II^–**5**(H_2_O)](ClO_4_)_2_} complex. Therefore, the (9-anthracen­yl)methyl pendant arm does not completely inhibit the chelation of the zinc(II) ion but reduces the basicity of the bound nitro­gen atom; therefore, the polyamine ring of Ant-[12]aneN3 cannot chelate zinc(II). The protonation of the secondary nitro­gen atoms may be related to the synthesis conditions of the complex, which will be the subject of a future study. Ant-[12]aneN3 and its analogs exhibit DNA photocleavage activity (Ichimaru *et al.*, 2025 ▸). Thus, the crystal structure of the title compound will contribute to the design of polyamine derivatives with DNA photocleavage activity.

## Synthesis and crystallization

Ant-[12]aneN3 was synthesized using a previously reported synthetic method (Ichimaru *et al.*, 2025 ▸). Then, Ant-[12]aneN3 (72.4 mg, 0.20 mmol) was dissolved in MeOH (10 ml), and a MeOH solution of ZnCl_2_ (27.2 mg, 0.20 mmol, 1.0 eq.) was added dropwise. The reaction mixture was stirred at 296 K for 30 min. Subsequently, the particles in the reaction mixture were filtered out and the filtrate was allowed to stand at 296 K. Colorless crystals suitable for X-ray crystallography were obtained (12.6 mg).

## Refinement

Crystal data, data collection and structure refinement details are summarized in Table 2 ▸.

## Supplementary Material

Crystal structure: contains datablock(s) I. DOI: 10.1107/S2414314625003566/bx4034sup1.cif

Structure factors: contains datablock(s) I. DOI: 10.1107/S2414314625003566/bx4034Isup2.hkl

CCDC reference: 2445226

Additional supporting information:  crystallographic information; 3D view; checkCIF report

## Figures and Tables

**Table 1:** Hydrogen-bond geometry (Å, °)

*D*—H⋯*A*	*D*—H	H⋯*A*	*D*⋯*A*	*D*—H⋯*A*
N2—H2*A*⋯Cl1	0.88 (3)	2.39 (3)	3.273 (2)	176.6 (16)
N5—H5*A*⋯Cl1	0.88 (3)	2.42 (3)	3.227 (2)	153 (3)
N2—H2*B*⋯N1	0.96 (3)	2.21 (3)	2.854 (2)	124 (2)
N2—H2*B*⋯N3	0.96 (3)	2.05 (3)	2.830 (3)	137 (2)
N5—H5*B*⋯N4	1.00 (4)	2.59 (3)	3.194 (3)	120 (2)
N5—H5*B*⋯N6	0.99 (4)	1.77 (3)	2.644 (3)	145 (3)

**Table 2:** Experimental details

Crystal data	
Chemical formula	(C_24_H_32_N_3_)_2_[ZnCl_4_]
*M*_r_	932.22
Crystal system, space group	Monoclinic, *P*2_1_/*c*
Temperature (K)	100
*a*, *b*, *c* (Å)	14.0683 (2), 18.4885 (2), 17.7569 (2)
β (°)	93.407 (1)
*V* (Å^3^)	4610.44 (10)
*Z*	4
Radiation type	Cu *K*α
μ (mm^−1^)	3.18
Crystal size (mm)	0.69 × 0.58 × 0.37

Data collection	
Diffractometer	Rigaku XtaLAB Synergy-i
Absorption correction	Multi-scan (*CrysAlis PRO*; Rigaku OD, 2022 ▸)
*T*_min_, *T*_max_	0.332, 1.000
No. of measured, independent and observed [*I* > 2σ(*I*)] reflections	47324, 8409, 7651
*R*_int_	0.119
(sin θ/λ)_max_ (Å^−1^)	0.602

Refinement	
*R*[*F*^2^ > 2σ(*F*^2^)], *wR*(*F*^2^), *S*	0.050, 0.138, 1.03
No. of reflections	8409
No. of parameters	552
H-atom treatment	H atoms treated by a mixture of independent and constrained refinement
Δρ_max_, Δρ_min_ (e Å^−3^)	0.86, −1.13

Computer programs: *CrysAlis PRO* (Rigaku OD, 2022 ▸), *SHELXT2018/2* (Sheldrick, 2015 ▸*a* ▸), *SHELXL2019/3* (Sheldrick, 2015*b* ▸) and *OLEX2* (Dolomanov *et al.*, 2009 ▸).

## full crystallographic data

### Bis[5-(anthracen-9-ylmethyl)-1,5,9-triazacyclododecan-1-ium] tetrachloridozincate Crystal data

**Table:**

(C_24_H_32_N_3_)_2_[ZnCl_4_]	*F*(000) = 1968
*M**_r_* = 932.22	*D*_x_ = 1.343 Mg m^−^^3^
Monoclinic, *P*2_1_/*c*	Cu *K*α radiation, λ = 1.54184 Å
*a* = 14.0683 (2) Å	Cell parameters from 30005 reflections
*b* = 18.4885 (2) Å	θ = 2.5–68.2°
*c* = 17.7569 (2) Å	µ = 3.18 mm^−^^1^
β = 93.407 (1)°	*T* = 100 K
*V* = 4610.44 (10) Å^3^	Block, colourless
*Z* = 4	0.69 × 0.58 × 0.37 mm

### Bis[5-(anthracen-9-ylmethyl)-1,5,9-triazacyclododecan-1-ium] tetrachloridozincate Data collection

**Table:**

Rigaku XtaLAB Synergy-i diffractometer	7651 reflections with *I* > 2σ(*I*)
Detector resolution: 10.0 pixels mm^-1^	*R*_int_ = 0.119
ω scans	θ_max_ = 68.3°, θ_min_ = 3.2°
Absorption correction: multi-scan (CrysAlisPro; Rigaku OD, 2022)	*h* = −16→16
*T*_min_ = 0.332, *T*_max_ = 1.000	*k* = −22→22
47324 measured reflections	*l* = −21→21
8409 independent reflections	

### Bis[5-(anthracen-9-ylmethyl)-1,5,9-triazacyclododecan-1-ium] tetrachloridozincate Refinement

**Table:**

Refinement on *F*^2^	0 restraints
Least-squares matrix: full	Hydrogen site location: mixed
*R*[*F*^2^ > 2σ(*F*^2^)] = 0.050	H atoms treated by a mixture of independent and constrained refinement
*wR*(*F*^2^) = 0.138	*w* = 1/[σ^2^(*F*_o_^2^) + (0.0947*P*)^2^ + 1.017*P*] where *P* = (*F*_o_^2^ + 2*F*_c_^2^)/3
*S* = 1.03	(Δ/σ)_max_ = 0.001
8409 reflections	Δρ_max_ = 0.86 e Å^−^^3^
552 parameters	Δρ_min_ = −1.13 e Å^−^^3^

### Bis[5-(anthracen-9-ylmethyl)-1,5,9-triazacyclododecan-1-ium] tetrachloridozincate Special details

**Table:**

Geometry. All esds (except the esd in the dihedral angle between two l.s. planes) are estimated using the full covariance matrix. The cell esds are taken into account individually in the estimation of esds in distances, angles and torsion angles; correlations between esds in cell parameters are only used when they are defined by crystal symmetry. An approximate (isotropic) treatment of cell esds is used for estimating esds involving l.s. planes.
Refinement. All hydrogen atoms were located by a geometrical calculation, and were not refined.

### Bis[5-(anthracen-9-ylmethyl)-1,5,9-triazacyclododecan-1-ium] tetrachloridozincate Fractional atomic coordinates and isotropic or equivalent isotropic displacement parameters (Å^2^)

**Table:**

	*x*	*y*	*z*	*U*_iso_*/*U*_eq_	
Zn1	0.46001 (2)	0.20693 (2)	0.19895 (2)	0.01677 (11)	
Cl1	0.55113 (3)	0.31066 (3)	0.18017 (3)	0.01971 (14)	
Cl2	0.54601 (4)	0.14222 (3)	0.28563 (3)	0.02533 (15)	
Cl3	0.42573 (4)	0.15296 (3)	0.08674 (3)	0.02855 (15)	
Cl4	0.32647 (4)	0.25051 (4)	0.24458 (4)	0.03857 (18)	
N1	0.78988 (11)	0.40868 (9)	0.34143 (10)	0.0153 (4)	
N2	0.62966 (12)	0.31644 (10)	0.35733 (11)	0.0167 (4)	
H2A	0.6069 (19)	0.3133 (14)	0.3101 (17)	0.020*	
N4	0.16050 (12)	0.44481 (10)	0.13858 (10)	0.0191 (4)	
N3	0.58552 (13)	0.46271 (11)	0.38661 (11)	0.0218 (4)	
H3	0.6011 (19)	0.5012 (15)	0.4113 (16)	0.026*	
N5	0.37799 (14)	0.40949 (11)	0.11131 (12)	0.0248 (4)	
H5A	0.412 (2)	0.3820 (16)	0.1427 (17)	0.030*	
C8	1.05304 (15)	0.38679 (11)	0.52527 (12)	0.0177 (4)	
C3	1.00339 (14)	0.41464 (11)	0.45784 (12)	0.0164 (4)	
N6	0.25947 (16)	0.31225 (11)	0.04973 (13)	0.0311 (5)	
H6A	0.280 (2)	0.2656 (17)	0.0744 (17)	0.037*	
C27	0.12560 (14)	0.57593 (12)	0.02968 (12)	0.0168 (4)	
C17	0.78007 (15)	0.27801 (12)	0.30566 (12)	0.0196 (4)	
H17A	0.742780	0.284477	0.257072	0.024*	
H17B	0.820600	0.234627	0.301190	0.024*	
C9	1.00241 (15)	0.36980 (11)	0.58776 (12)	0.0183 (4)	
H9	1.035900	0.352497	0.632187	0.022*	
C32	0.12851 (14)	0.65100 (12)	0.01011 (12)	0.0178 (4)	
C33	0.07136 (15)	0.70005 (12)	0.04626 (13)	0.0191 (4)	
H33	0.072757	0.749748	0.032763	0.023*	
C34	0.01216 (14)	0.67736 (12)	0.10192 (12)	0.0178 (4)	
C26	0.06735 (14)	0.55201 (11)	0.08693 (12)	0.0167 (4)	
C15	0.85342 (14)	0.40437 (11)	0.51996 (12)	0.0166 (4)	
C10	0.90386 (15)	0.37747 (11)	0.58698 (12)	0.0182 (4)	
C7	1.15404 (15)	0.37664 (12)	0.52681 (13)	0.0205 (5)	
H7	1.186529	0.358397	0.571253	0.025*	
C18	0.71181 (14)	0.26554 (12)	0.36792 (13)	0.0191 (4)	
H18A	0.688588	0.214989	0.366044	0.023*	
H18B	0.745279	0.273759	0.417812	0.023*	
C40	0.20365 (15)	0.49277 (12)	0.19707 (12)	0.0211 (5)	
H40A	0.167530	0.488501	0.243042	0.025*	
H40B	0.198152	0.543422	0.179265	0.025*	
C6	1.20429 (15)	0.39260 (12)	0.46599 (14)	0.0233 (5)	
H6	1.271325	0.385754	0.468051	0.028*	
C16	0.84377 (14)	0.34406 (12)	0.32025 (12)	0.0193 (4)	
H16A	0.892254	0.332690	0.361237	0.023*	
H16B	0.877559	0.354842	0.274219	0.023*	
C4	1.06014 (16)	0.43059 (12)	0.39552 (13)	0.0214 (5)	
H4	1.030037	0.449344	0.350350	0.026*	
C39	0.00988 (14)	0.60255 (12)	0.12308 (12)	0.0178 (4)	
C5	1.15606 (16)	0.41958 (13)	0.39925 (14)	0.0249 (5)	
H5	1.191243	0.430122	0.356554	0.030*	
C1	0.84957 (14)	0.45874 (12)	0.38912 (12)	0.0184 (4)	
H1A	0.808182	0.497413	0.407569	0.022*	
H1B	0.896243	0.481913	0.357203	0.022*	
C28	0.18310 (15)	0.52714 (13)	−0.01105 (13)	0.0230 (5)	
H28	0.181781	0.476861	−0.000126	0.028*	
C11	0.85266 (16)	0.35898 (12)	0.65105 (12)	0.0211 (5)	
H11	0.886482	0.341973	0.695456	0.025*	
C14	0.75149 (15)	0.41009 (12)	0.52152 (13)	0.0198 (4)	
H14	0.715822	0.427661	0.478224	0.024*	
C24	0.75139 (15)	0.44651 (13)	0.27301 (12)	0.0208 (5)	
H24A	0.720427	0.410091	0.238828	0.025*	
H24B	0.805675	0.466880	0.247003	0.025*	
C2	0.90331 (15)	0.42452 (11)	0.45650 (12)	0.0171 (4)	
C25	0.06714 (15)	0.47286 (12)	0.10799 (13)	0.0221 (5)	
H25A	0.019521	0.465160	0.146063	0.026*	
H25B	0.046666	0.444344	0.062697	0.026*	
C23	0.67998 (15)	0.50741 (12)	0.28372 (13)	0.0229 (5)	
H23A	0.707626	0.540917	0.322612	0.028*	
H23B	0.671824	0.534822	0.235875	0.028*	
C13	0.70502 (15)	0.39089 (12)	0.58360 (13)	0.0230 (5)	
H13	0.637615	0.394703	0.582601	0.028*	
C22	0.58215 (15)	0.48423 (12)	0.30667 (13)	0.0229 (5)	
H22A	0.536715	0.524729	0.298272	0.027*	
H22B	0.559228	0.443081	0.274863	0.027*	
C41	0.30848 (15)	0.47594 (12)	0.21743 (13)	0.0224 (5)	
H41A	0.333025	0.510583	0.256417	0.027*	
H41B	0.313504	0.426768	0.239370	0.027*	
C19	0.54923 (16)	0.30574 (12)	0.40748 (13)	0.0217 (5)	
H19A	0.572372	0.312149	0.460812	0.026*	
H19B	0.524010	0.255965	0.401348	0.026*	
C42	0.37033 (16)	0.48012 (12)	0.14998 (14)	0.0247 (5)	
H42A	0.342912	0.516192	0.113654	0.030*	
H42B	0.434843	0.496813	0.167280	0.030*	
C31	0.18959 (16)	0.67461 (14)	−0.04690 (13)	0.0248 (5)	
H31	0.191748	0.724407	−0.059867	0.030*	
C30	0.24436 (17)	0.62644 (15)	−0.08251 (13)	0.0300 (5)	
H30	0.285970	0.642839	−0.119129	0.036*	
C48	0.14675 (17)	0.37239 (12)	0.17049 (14)	0.0264 (5)	
H48A	0.102066	0.376442	0.211299	0.032*	
H48B	0.208517	0.355368	0.193596	0.032*	
C29	0.23957 (17)	0.55202 (15)	−0.06519 (13)	0.0297 (5)	
H29	0.276435	0.518632	−0.091806	0.036*	
C21	0.49365 (16)	0.43702 (13)	0.41228 (13)	0.0251 (5)	
H21A	0.442352	0.469514	0.392019	0.030*	
H21B	0.495189	0.439670	0.468020	0.030*	
C35	−0.04748 (15)	0.72853 (13)	0.13690 (14)	0.0240 (5)	
H35	−0.044708	0.778104	0.123145	0.029*	
C38	−0.05485 (15)	0.58351 (14)	0.17951 (13)	0.0243 (5)	
H38	−0.058174	0.534624	0.195604	0.029*	
C12	0.75582 (16)	0.36524 (12)	0.64987 (13)	0.0237 (5)	
H12	0.722666	0.352532	0.693034	0.028*	
C20	0.47057 (15)	0.35996 (13)	0.38765 (13)	0.0253 (5)	
H20A	0.412100	0.344286	0.411429	0.030*	
H20B	0.456840	0.359443	0.332317	0.030*	
C37	−0.11152 (16)	0.63378 (15)	0.21047 (14)	0.0303 (6)	
H37	−0.154334	0.619112	0.246967	0.036*	
C47	0.10856 (19)	0.31524 (13)	0.11422 (16)	0.0331 (6)	
H47A	0.116035	0.267111	0.138281	0.040*	
H47B	0.039524	0.323683	0.103886	0.040*	
C36	−0.10783 (17)	0.70738 (15)	0.18932 (15)	0.0306 (6)	
H36	−0.147488	0.741841	0.211757	0.037*	
C44	0.4117 (2)	0.34162 (15)	−0.00535 (15)	0.0367 (6)	
H44A	0.445088	0.302985	0.024402	0.044*	
H44B	0.442434	0.345915	−0.053895	0.044*	
C43	0.42329 (18)	0.41288 (14)	0.03737 (15)	0.0324 (6)	
H43A	0.491896	0.423946	0.046224	0.039*	
H43B	0.393744	0.452343	0.006416	0.039*	
C46	0.15522 (19)	0.31325 (13)	0.03995 (16)	0.0336 (6)	
H46A	0.133398	0.269654	0.011539	0.040*	
H46B	0.134975	0.356188	0.009777	0.040*	
C45	0.3076 (2)	0.31969 (15)	−0.02110 (16)	0.0388 (7)	
H45A	0.274505	0.356748	−0.053256	0.047*	
H45B	0.304482	0.273162	−0.048731	0.047*	
H2B	0.6497 (19)	0.3654 (15)	0.3679 (15)	0.027 (7)*	
H5B	0.316 (3)	0.3846 (18)	0.1000 (19)	0.050 (9)*	

### Bis[5-(anthracen-9-ylmethyl)-1,5,9-triazacyclododecan-1-ium] tetrachloridozincate Atomic displacement parameters (Å^2^)

**Table:**

	*U*^11^	*U*^22^	*U*^33^	*U*^12^	*U*^13^	*U*^23^
Zn1	0.00583 (17)	0.02313 (18)	0.02092 (18)	−0.00015 (10)	−0.00275 (12)	0.00075 (11)
Cl1	0.0113 (3)	0.0272 (3)	0.0203 (3)	−0.00513 (18)	−0.00231 (19)	0.00106 (19)
Cl2	0.0225 (3)	0.0232 (3)	0.0285 (3)	0.0029 (2)	−0.0130 (2)	0.0000 (2)
Cl3	0.0288 (3)	0.0325 (3)	0.0231 (3)	−0.0076 (2)	−0.0086 (2)	−0.0018 (2)
Cl4	0.0118 (3)	0.0508 (4)	0.0543 (4)	0.0088 (2)	0.0114 (3)	0.0089 (3)
N1	0.0086 (8)	0.0215 (9)	0.0158 (8)	0.0000 (7)	−0.0012 (6)	0.0000 (7)
N2	0.0090 (9)	0.0244 (10)	0.0163 (9)	−0.0008 (7)	−0.0020 (7)	−0.0011 (7)
N4	0.0133 (9)	0.0207 (9)	0.0221 (9)	−0.0003 (7)	−0.0085 (7)	0.0023 (7)
N3	0.0172 (9)	0.0255 (10)	0.0222 (10)	0.0029 (8)	−0.0036 (7)	−0.0023 (8)
N5	0.0188 (10)	0.0267 (10)	0.0282 (11)	0.0045 (8)	−0.0049 (8)	−0.0022 (9)
C8	0.0137 (10)	0.0183 (10)	0.0208 (11)	−0.0005 (8)	−0.0023 (8)	−0.0047 (8)
C3	0.0124 (10)	0.0182 (10)	0.0184 (10)	−0.0036 (8)	−0.0015 (8)	−0.0036 (8)
N6	0.0337 (12)	0.0259 (11)	0.0319 (12)	0.0046 (9)	−0.0121 (9)	−0.0027 (9)
C27	0.0065 (9)	0.0288 (11)	0.0147 (10)	0.0014 (8)	−0.0040 (7)	−0.0018 (8)
C17	0.0101 (10)	0.0259 (11)	0.0222 (11)	0.0028 (8)	−0.0047 (8)	−0.0070 (9)
C9	0.0146 (10)	0.0218 (11)	0.0177 (10)	0.0026 (8)	−0.0070 (8)	−0.0020 (8)
C32	0.0086 (10)	0.0295 (12)	0.0149 (10)	−0.0015 (8)	−0.0025 (8)	0.0012 (8)
C33	0.0121 (10)	0.0240 (11)	0.0203 (11)	−0.0023 (8)	−0.0056 (8)	0.0003 (9)
C34	0.0067 (9)	0.0267 (11)	0.0195 (10)	0.0000 (8)	−0.0041 (8)	−0.0027 (9)
C26	0.0073 (9)	0.0245 (11)	0.0174 (10)	−0.0004 (8)	−0.0065 (7)	0.0014 (8)
C15	0.0126 (10)	0.0189 (10)	0.0181 (10)	−0.0006 (8)	−0.0022 (8)	−0.0041 (8)
C10	0.0159 (10)	0.0187 (10)	0.0198 (10)	0.0002 (8)	−0.0016 (8)	−0.0045 (8)
C7	0.0146 (11)	0.0217 (11)	0.0246 (11)	0.0015 (8)	−0.0039 (8)	−0.0034 (9)
C18	0.0104 (10)	0.0216 (11)	0.0247 (11)	−0.0005 (8)	−0.0043 (8)	−0.0009 (9)
C40	0.0175 (11)	0.0249 (11)	0.0201 (11)	0.0003 (9)	−0.0049 (8)	−0.0007 (9)
C6	0.0106 (10)	0.0282 (12)	0.0311 (12)	−0.0006 (8)	0.0001 (8)	−0.0042 (10)
C16	0.0094 (10)	0.0285 (11)	0.0199 (10)	−0.0005 (8)	0.0008 (8)	−0.0034 (9)
C4	0.0179 (11)	0.0253 (11)	0.0208 (11)	−0.0046 (8)	−0.0011 (8)	−0.0004 (9)
C39	0.0061 (9)	0.0299 (11)	0.0168 (10)	−0.0013 (8)	−0.0051 (7)	0.0001 (9)
C5	0.0176 (11)	0.0301 (12)	0.0276 (12)	−0.0046 (9)	0.0057 (9)	−0.0016 (10)
C1	0.0134 (10)	0.0231 (11)	0.0181 (10)	−0.0022 (8)	−0.0044 (8)	−0.0006 (8)
C28	0.0174 (11)	0.0305 (12)	0.0204 (11)	0.0063 (9)	−0.0044 (8)	−0.0022 (9)
C11	0.0198 (11)	0.0246 (11)	0.0188 (11)	0.0009 (8)	−0.0006 (9)	−0.0012 (9)
C14	0.0136 (10)	0.0240 (11)	0.0212 (11)	0.0011 (8)	−0.0029 (8)	−0.0033 (9)
C24	0.0144 (10)	0.0315 (12)	0.0158 (10)	−0.0030 (9)	−0.0029 (8)	0.0025 (9)
C2	0.0133 (10)	0.0189 (10)	0.0187 (10)	−0.0028 (8)	−0.0030 (8)	−0.0037 (8)
C25	0.0117 (10)	0.0253 (11)	0.0284 (12)	−0.0017 (8)	−0.0062 (8)	0.0031 (9)
C23	0.0202 (11)	0.0240 (11)	0.0238 (11)	−0.0014 (9)	−0.0056 (9)	0.0058 (9)
C13	0.0128 (10)	0.0292 (12)	0.0268 (12)	0.0000 (9)	0.0006 (8)	−0.0059 (9)
C22	0.0171 (11)	0.0247 (11)	0.0261 (12)	0.0056 (9)	−0.0044 (9)	0.0026 (9)
C41	0.0178 (11)	0.0227 (11)	0.0253 (11)	−0.0001 (8)	−0.0104 (9)	−0.0020 (9)
C19	0.0148 (11)	0.0269 (11)	0.0237 (11)	−0.0037 (8)	0.0040 (9)	−0.0021 (9)
C42	0.0146 (11)	0.0251 (11)	0.0332 (13)	0.0008 (9)	−0.0077 (9)	−0.0035 (10)
C31	0.0198 (11)	0.0357 (13)	0.0188 (11)	−0.0056 (10)	0.0014 (9)	0.0048 (10)
C30	0.0188 (12)	0.0525 (16)	0.0193 (11)	−0.0043 (11)	0.0050 (9)	0.0005 (11)
C48	0.0229 (12)	0.0258 (12)	0.0293 (12)	−0.0006 (9)	−0.0089 (9)	0.0064 (10)
C29	0.0193 (12)	0.0505 (16)	0.0194 (11)	0.0099 (10)	0.0016 (9)	−0.0047 (11)
C21	0.0156 (11)	0.0344 (12)	0.0254 (12)	0.0065 (9)	0.0022 (9)	−0.0030 (10)
C35	0.0108 (10)	0.0296 (12)	0.0309 (12)	0.0003 (9)	−0.0045 (9)	−0.0075 (10)
C38	0.0138 (11)	0.0363 (13)	0.0229 (11)	−0.0069 (9)	0.0020 (9)	0.0036 (10)
C12	0.0210 (11)	0.0286 (12)	0.0221 (11)	0.0010 (9)	0.0054 (9)	−0.0021 (9)
C20	0.0090 (10)	0.0432 (14)	0.0238 (11)	0.0015 (9)	0.0025 (8)	−0.0052 (10)
C37	0.0127 (11)	0.0551 (16)	0.0234 (12)	−0.0068 (10)	0.0037 (9)	−0.0056 (11)
C47	0.0283 (14)	0.0234 (12)	0.0452 (16)	−0.0044 (10)	−0.0178 (11)	0.0043 (11)
C36	0.0122 (11)	0.0476 (15)	0.0320 (13)	0.0008 (10)	0.0016 (10)	−0.0160 (11)
C44	0.0382 (15)	0.0404 (15)	0.0312 (13)	0.0157 (12)	0.0004 (11)	−0.0021 (12)
C43	0.0255 (13)	0.0375 (14)	0.0341 (14)	0.0078 (11)	0.0026 (10)	0.0018 (11)
C46	0.0354 (15)	0.0243 (12)	0.0384 (15)	0.0008 (10)	−0.0199 (12)	−0.0051 (11)
C45	0.0469 (17)	0.0366 (14)	0.0319 (14)	0.0092 (12)	−0.0064 (12)	−0.0101 (12)

### Bis[5-(anthracen-9-ylmethyl)-1,5,9-triazacyclododecan-1-ium] tetrachloridozincate Geometric parameters (Å, º)

**Table:**

Zn1—Cl1	2.3416 (5)	C5—H5	0.9500
Zn1—Cl2	2.2456 (6)	C1—H1A	0.9900
Zn1—Cl3	2.2550 (6)	C1—H1B	0.9900
Zn1—Cl4	2.2396 (6)	C1—C2	1.515 (3)
N1—C16	1.476 (3)	C28—H28	0.9500
N1—C1	1.481 (3)	C28—C29	1.363 (3)
N1—C24	1.477 (3)	C11—H11	0.9500
N2—H2A	0.88 (3)	C11—C12	1.366 (3)
N2—C18	1.494 (3)	C14—H14	0.9500
N2—C19	1.494 (3)	C14—C13	1.362 (3)
N2—H2B	0.96 (3)	C24—H24A	0.9900
N4—C40	1.469 (3)	C24—H24B	0.9900
N4—C25	1.485 (3)	C24—C23	1.528 (3)
N4—C48	1.471 (3)	C25—H25A	0.9900
N3—H3	0.86 (3)	C25—H25B	0.9900
N3—C22	1.472 (3)	C23—H23A	0.9900
N3—C21	1.474 (3)	C23—H23B	0.9900
N5—H5A	0.87 (3)	C23—C22	1.520 (3)
N5—C42	1.482 (3)	C13—H13	0.9500
N5—C43	1.494 (3)	C13—C12	1.422 (3)
N5—H5B	0.99 (4)	C22—H22A	0.9900
C8—C3	1.445 (3)	C22—H22B	0.9900
C8—C9	1.390 (3)	C41—H41A	0.9900
C8—C7	1.432 (3)	C41—H41B	0.9900
C3—C4	1.433 (3)	C41—C42	1.524 (3)
C3—C2	1.419 (3)	C19—H19A	0.9900
N6—H6A	1.00 (3)	C19—H19B	0.9900
N6—C46	1.467 (3)	C19—C20	1.519 (3)
N6—C45	1.470 (4)	C42—H42A	0.9900
C27—C32	1.432 (3)	C42—H42B	0.9900
C27—C26	1.414 (3)	C31—H31	0.9500
C27—C28	1.436 (3)	C31—C30	1.358 (4)
C17—H17A	0.9900	C30—H30	0.9500
C17—H17B	0.9900	C30—C29	1.413 (4)
C17—C18	1.524 (3)	C48—H48A	0.9900
C17—C16	1.528 (3)	C48—H48B	0.9900
C9—H9	0.9500	C48—C47	1.530 (3)
C9—C10	1.393 (3)	C29—H29	0.9500
C32—C33	1.394 (3)	C21—H21A	0.9900
C32—C31	1.434 (3)	C21—H21B	0.9900
C33—H33	0.9500	C21—C20	1.520 (3)
C33—C34	1.394 (3)	C35—H35	0.9500
C34—C39	1.434 (3)	C35—C36	1.354 (4)
C34—C35	1.431 (3)	C38—H38	0.9500
C26—C39	1.414 (3)	C38—C37	1.362 (4)
C26—C25	1.510 (3)	C12—H12	0.9500
C15—C10	1.438 (3)	C20—H20A	0.9900
C15—C14	1.440 (3)	C20—H20B	0.9900
C15—C2	1.413 (3)	C37—H37	0.9500
C10—C11	1.424 (3)	C37—C36	1.413 (4)
C7—H7	0.9500	C47—H47A	0.9900
C7—C6	1.358 (3)	C47—H47B	0.9900
C18—H18A	0.9900	C47—C46	1.508 (4)
C18—H18B	0.9900	C36—H36	0.9500
C40—H40A	0.9900	C44—H44A	0.9900
C40—H40B	0.9900	C44—H44B	0.9900
C40—C41	1.529 (3)	C44—C43	1.524 (4)
C6—H6	0.9500	C44—C45	1.530 (4)
C6—C5	1.421 (3)	C43—H43A	0.9900
C16—H16A	0.9900	C43—H43B	0.9900
C16—H16B	0.9900	C46—H46A	0.9900
C4—H4	0.9500	C46—H46B	0.9900
C4—C5	1.362 (3)	C45—H45A	0.9900
C39—C38	1.437 (3)	C45—H45B	0.9900
			
Cl2—Zn1—Cl1	104.88 (2)	H24A—C24—H24B	107.2
Cl2—Zn1—Cl3	116.62 (2)	C23—C24—H24A	108.0
Cl3—Zn1—Cl1	109.07 (2)	C23—C24—H24B	108.0
Cl4—Zn1—Cl1	103.59 (2)	C3—C2—C1	121.01 (19)
Cl4—Zn1—Cl2	111.91 (3)	C15—C2—C3	119.50 (19)
Cl4—Zn1—Cl3	109.81 (3)	C15—C2—C1	119.46 (18)
C16—N1—C1	111.67 (15)	N4—C25—C26	114.48 (17)
C16—N1—C24	110.04 (16)	N4—C25—H25A	108.6
C24—N1—C1	110.00 (16)	N4—C25—H25B	108.6
H2A—N2—H2B	109 (2)	C26—C25—H25A	108.6
C18—N2—H2A	108.2 (17)	C26—C25—H25B	108.6
C18—N2—C19	116.70 (18)	H25A—C25—H25B	107.6
C18—N2—H2B	110.6 (16)	C24—C23—H23A	108.3
C19—N2—H2A	108.2 (17)	C24—C23—H23B	108.3
C19—N2—H2B	103.3 (17)	H23A—C23—H23B	107.4
C40—N4—C25	111.55 (17)	C22—C23—C24	115.97 (19)
C40—N4—C48	109.59 (17)	C22—C23—H23A	108.3
C48—N4—C25	108.98 (17)	C22—C23—H23B	108.3
C22—N3—H3	105.1 (19)	C14—C13—H13	119.5
C22—N3—C21	113.99 (17)	C14—C13—C12	121.0 (2)
C21—N3—H3	108.2 (19)	C12—C13—H13	119.5
H5A—N5—H5B	107 (3)	N3—C22—C23	110.91 (18)
C42—N5—H5A	105.6 (19)	N3—C22—H22A	109.5
C42—N5—C43	114.6 (2)	N3—C22—H22B	109.5
C42—N5—H5B	114.6 (19)	C23—C22—H22A	109.5
C43—N5—H5A	109.9 (19)	C23—C22—H22B	109.5
C43—N5—H5B	105 (2)	H22A—C22—H22B	108.0
C9—C8—C3	119.79 (19)	C40—C41—H41A	109.0
C9—C8—C7	120.67 (19)	C40—C41—H41B	109.0
C7—C8—C3	119.5 (2)	H41A—C41—H41B	107.8
C4—C3—C8	116.70 (19)	C42—C41—C40	113.05 (18)
C2—C3—C8	119.36 (19)	C42—C41—H41A	109.0
C2—C3—C4	123.9 (2)	C42—C41—H41B	109.0
C46—N6—H6A	108.8 (17)	N2—C19—H19A	109.7
C46—N6—C45	113.9 (2)	N2—C19—H19B	109.7
C45—N6—H6A	108.6 (18)	N2—C19—C20	109.97 (18)
C32—C27—C28	117.50 (19)	H19A—C19—H19B	108.2
C26—C27—C32	120.25 (19)	C20—C19—H19A	109.7
C26—C27—C28	122.2 (2)	C20—C19—H19B	109.7
H17A—C17—H17B	107.8	N5—C42—C41	112.34 (19)
C18—C17—H17A	109.0	N5—C42—H42A	109.1
C18—C17—H17B	109.0	N5—C42—H42B	109.1
C18—C17—C16	112.73 (18)	C41—C42—H42A	109.1
C16—C17—H17A	109.0	C41—C42—H42B	109.1
C16—C17—H17B	109.0	H42A—C42—H42B	107.9
C8—C9—H9	119.1	C32—C31—H31	119.7
C8—C9—C10	121.74 (19)	C30—C31—C32	120.6 (2)
C10—C9—H9	119.1	C30—C31—H31	119.7
C27—C32—C31	119.6 (2)	C31—C30—H30	119.9
C33—C32—C27	119.57 (19)	C31—C30—C29	120.1 (2)
C33—C32—C31	120.9 (2)	C29—C30—H30	119.9
C32—C33—H33	119.5	N4—C48—H48A	108.5
C32—C33—C34	121.0 (2)	N4—C48—H48B	108.5
C34—C33—H33	119.5	N4—C48—C47	115.2 (2)
C33—C34—C39	120.0 (2)	H48A—C48—H48B	107.5
C33—C34—C35	119.9 (2)	C47—C48—H48A	108.5
C35—C34—C39	120.1 (2)	C47—C48—H48B	108.5
C27—C26—C39	119.40 (19)	C28—C29—C30	121.3 (2)
C27—C26—C25	119.39 (19)	C28—C29—H29	119.3
C39—C26—C25	121.22 (19)	C30—C29—H29	119.3
C10—C15—C14	116.9 (2)	N3—C21—H21A	109.0
C2—C15—C10	120.49 (19)	N3—C21—H21B	109.0
C2—C15—C14	122.59 (19)	N3—C21—C20	112.91 (18)
C9—C10—C15	119.0 (2)	H21A—C21—H21B	107.8
C9—C10—C11	121.20 (19)	C20—C21—H21A	109.0
C11—C10—C15	119.75 (19)	C20—C21—H21B	109.0
C8—C7—H7	119.4	C34—C35—H35	119.5
C6—C7—C8	121.3 (2)	C36—C35—C34	121.0 (2)
C6—C7—H7	119.4	C36—C35—H35	119.5
N2—C18—C17	109.24 (17)	C39—C38—H38	119.2
N2—C18—H18A	109.8	C37—C38—C39	121.7 (2)
N2—C18—H18B	109.8	C37—C38—H38	119.2
C17—C18—H18A	109.8	C11—C12—C13	119.5 (2)
C17—C18—H18B	109.8	C11—C12—H12	120.2
H18A—C18—H18B	108.3	C13—C12—H12	120.2
N4—C40—H40A	108.9	C19—C20—C21	114.31 (18)
N4—C40—H40B	108.9	C19—C20—H20A	108.7
N4—C40—C41	113.36 (18)	C19—C20—H20B	108.7
H40A—C40—H40B	107.7	C21—C20—H20A	108.7
C41—C40—H40A	108.9	C21—C20—H20B	108.7
C41—C40—H40B	108.9	H20A—C20—H20B	107.6
C7—C6—H6	120.2	C38—C37—H37	119.4
C7—C6—C5	119.6 (2)	C38—C37—C36	121.2 (2)
C5—C6—H6	120.2	C36—C37—H37	119.4
N1—C16—C17	112.66 (17)	C48—C47—H47A	108.4
N1—C16—H16A	109.1	C48—C47—H47B	108.4
N1—C16—H16B	109.1	H47A—C47—H47B	107.4
C17—C16—H16A	109.1	C46—C47—C48	115.7 (2)
C17—C16—H16B	109.1	C46—C47—H47A	108.4
H16A—C16—H16B	107.8	C46—C47—H47B	108.4
C3—C4—H4	119.1	C35—C36—C37	119.7 (2)
C5—C4—C3	121.8 (2)	C35—C36—H36	120.2
C5—C4—H4	119.1	C37—C36—H36	120.2
C34—C39—C38	116.4 (2)	H44A—C44—H44B	107.8
C26—C39—C34	119.74 (19)	C43—C44—H44A	109.0
C26—C39—C38	123.9 (2)	C43—C44—H44B	109.0
C6—C5—H5	119.5	C43—C44—C45	113.1 (2)
C4—C5—C6	121.0 (2)	C45—C44—H44A	109.0
C4—C5—H5	119.5	C45—C44—H44B	109.0
N1—C1—H1A	108.4	N5—C43—C44	111.3 (2)
N1—C1—H1B	108.4	N5—C43—H43A	109.4
N1—C1—C2	115.45 (17)	N5—C43—H43B	109.4
H1A—C1—H1B	107.5	C44—C43—H43A	109.4
C2—C1—H1A	108.4	C44—C43—H43B	109.4
C2—C1—H1B	108.4	H43A—C43—H43B	108.0
C27—C28—H28	119.6	N6—C46—C47	112.4 (2)
C29—C28—C27	120.9 (2)	N6—C46—H46A	109.1
C29—C28—H28	119.6	N6—C46—H46B	109.1
C10—C11—H11	119.4	C47—C46—H46A	109.1
C12—C11—C10	121.3 (2)	C47—C46—H46B	109.1
C12—C11—H11	119.4	H46A—C46—H46B	107.9
C15—C14—H14	119.2	N6—C45—C44	110.7 (2)
C13—C14—C15	121.5 (2)	N6—C45—H45A	109.5
C13—C14—H14	119.2	N6—C45—H45B	109.5
N1—C24—H24A	108.0	C44—C45—H45A	109.5
N1—C24—H24B	108.0	C44—C45—H45B	109.5
N1—C24—C23	117.27 (18)	H45A—C45—H45B	108.1
			
N1—C1—C2—C3	107.8 (2)	C18—N2—C19—C20	−178.05 (18)
N1—C1—C2—C15	−74.4 (2)	C18—C17—C16—N1	−48.3 (2)
N1—C24—C23—C22	−70.9 (2)	C40—N4—C25—C26	47.8 (3)
N2—C19—C20—C21	−73.1 (2)	C40—N4—C48—C47	−175.9 (2)
N4—C40—C41—C42	57.9 (3)	C40—C41—C42—N5	−91.1 (2)
N4—C48—C47—C46	45.3 (3)	C16—N1—C1—C2	−50.4 (2)
N3—C21—C20—C19	52.1 (3)	C16—N1—C24—C23	170.11 (18)
C8—C3—C4—C5	0.5 (3)	C16—C17—C18—N2	78.9 (2)
C8—C3—C2—C15	−2.4 (3)	C4—C3—C2—C15	177.4 (2)
C8—C3—C2—C1	175.43 (18)	C4—C3—C2—C1	−4.7 (3)
C8—C9—C10—C15	−0.6 (3)	C39—C34—C35—C36	−1.1 (3)
C8—C9—C10—C11	179.2 (2)	C39—C26—C25—N4	−118.1 (2)
C8—C7—C6—C5	−0.3 (3)	C39—C38—C37—C36	−1.2 (4)
C3—C8—C9—C10	1.3 (3)	C1—N1—C16—C17	152.55 (18)
C3—C8—C7—C6	−0.1 (3)	C1—N1—C24—C23	−66.5 (2)
C3—C4—C5—C6	−0.9 (3)	C28—C27—C32—C33	177.76 (19)
C27—C32—C33—C34	0.8 (3)	C28—C27—C32—C31	−1.7 (3)
C27—C32—C31—C30	0.2 (3)	C28—C27—C26—C39	−177.80 (18)
C27—C26—C39—C34	−0.7 (3)	C28—C27—C26—C25	1.8 (3)
C27—C26—C39—C38	177.19 (19)	C14—C15—C10—C9	178.92 (19)
C27—C26—C25—N4	62.3 (3)	C14—C15—C10—C11	−0.9 (3)
C27—C28—C29—C30	0.7 (3)	C14—C15—C2—C3	−177.43 (19)
C9—C8—C3—C4	−179.67 (19)	C14—C15—C2—C1	4.7 (3)
C9—C8—C3—C2	0.2 (3)	C14—C13—C12—C11	−0.7 (3)
C9—C8—C7—C6	179.6 (2)	C24—N1—C16—C17	−85.0 (2)
C9—C10—C11—C12	−178.8 (2)	C24—N1—C1—C2	−172.84 (18)
C32—C27—C26—C39	1.9 (3)	C24—C23—C22—N3	74.9 (2)
C32—C27—C26—C25	−178.54 (17)	C2—C3—C4—C5	−179.4 (2)
C32—C27—C28—C29	1.3 (3)	C2—C15—C10—C9	−1.6 (3)
C32—C33—C34—C39	0.4 (3)	C2—C15—C10—C11	178.55 (19)
C32—C33—C34—C35	−178.52 (19)	C2—C15—C14—C13	−179.4 (2)
C32—C31—C30—C29	1.9 (3)	C25—N4—C40—C41	−166.87 (18)
C33—C32—C31—C30	−179.3 (2)	C25—N4—C48—C47	61.7 (3)
C33—C34—C39—C26	−0.4 (3)	C25—C26—C39—C34	179.72 (17)
C33—C34—C39—C38	−178.47 (19)	C25—C26—C39—C38	−2.4 (3)
C33—C34—C35—C36	177.8 (2)	C22—N3—C21—C20	77.9 (2)
C34—C39—C38—C37	0.7 (3)	C19—N2—C18—C17	172.51 (18)
C34—C35—C36—C37	0.6 (3)	C42—N5—C43—C44	−169.30 (19)
C26—C27—C32—C33	−1.9 (3)	C31—C32—C33—C34	−179.75 (19)
C26—C27—C32—C31	178.60 (19)	C31—C30—C29—C28	−2.3 (4)
C26—C27—C28—C29	−179.0 (2)	C48—N4—C40—C41	72.4 (2)
C26—C39—C38—C37	−177.3 (2)	C48—N4—C25—C26	168.92 (19)
C15—C10—C11—C12	1.0 (3)	C48—C47—C46—N6	49.2 (3)
C15—C14—C13—C12	0.8 (3)	C21—N3—C22—C23	−177.94 (19)
C10—C15—C14—C13	0.0 (3)	C35—C34—C39—C26	178.47 (18)
C10—C15—C2—C3	3.2 (3)	C35—C34—C39—C38	0.4 (3)
C10—C15—C2—C1	−174.74 (18)	C38—C37—C36—C35	0.5 (4)
C10—C11—C12—C13	−0.2 (3)	C43—N5—C42—C41	169.84 (18)
C7—C8—C3—C4	0.0 (3)	C43—C44—C45—N6	−59.0 (3)
C7—C8—C3—C2	179.92 (19)	C46—N6—C45—C44	162.4 (2)
C7—C8—C9—C10	−178.36 (19)	C45—N6—C46—C47	−172.6 (2)
C7—C6—C5—C4	0.8 (3)	C45—C44—C43—N5	58.6 (3)

### Bis[5-(anthracen-9-ylmethyl)-1,5,9-triazacyclododecan-1-ium] tetrachloridozincate Hydrogen-bond geometry (Å, º)

**Table:**

*D*—H···*A*	*D*—H	H···*A*	*D*···*A*	*D*—H···*A*
N2—H2*A*···Cl1	0.88 (3)	2.39 (3)	3.273 (2)	176.6 (16)
N5—H5*A*···Cl1	0.88 (3)	2.42 (3)	3.227 (2)	153 (3)
N2—H2*B*···N1	0.96 (3)	2.21 (3)	2.854 (2)	124 (2)
N2—H2*B*···N3	0.96 (3)	2.05 (3)	2.830 (3)	137 (2)
N5—H5*B*···N4	1.00 (4)	2.59 (3)	3.194 (3)	120 (2)
N5—H5*B*···N6	0.99 (4)	1.77 (3)	2.644 (3)	145 (3)
